# Cinematic rendering of ^18^F‐DCFPyL PET/CT fusion data in a patient with metastatic clear cell renal cell carcinoma

**DOI:** 10.1002/bco2.324

**Published:** 2024-02-04

**Authors:** Steven P. Rowe, Sebastian Krueger, Michael A. Gorin, Elliot K. Fishman

**Affiliations:** ^1^ Department of Radiology University of North Carolina Chapel Hill North Carolina USA; ^2^ Siemens Healthineers Erlanger Germany; ^3^ Milton and Carroll Petrie Department of Urology Icahn School of Medicine at Mount Sinai New York New York USA; ^4^ The Russell H. Morgan Department of Radiology and Radiological Science Johns Hopkins University School of Medicine Baltimore Maryland USA

**Keywords:** positron emission tomography, prostate‐specific membrane antigen, PSMA

Three‐dimensional visualizations of volumetric data are utilized for a variety of applications in medical imaging.^1^ Recently, a method known as cinematic rendering has been applied to standard acquisitions of medical image data to create photorealistic visualizations with high levels of surface detail.^2^, ^3^ The technique is based on complex path tracing that models the movement of millions of photons through a volume and includes information on how those photons interact with the matter in the volume.^4^ Generally, each type of tissue is assigned a colour and transparency based on a voxel histogram, and those characteristics of the tissue are then summed across the volume to create the rendering.^5^ This method has been most commonly applied to X‐ray computed tomography (CT), where it has been found to improve medical student education^6^ and the speed of surgical anatomic understanding.^7^

When working with fused dataset, such as CT in combination with position emission tomography (PET), the task of cinematic rendering is more complex, as there is nothing intrinsic to distinguish between the two image types within the visualization. To solve this, our group recently developed a method to include internal lighting for PET data so that it has a visually distinct signature from that of the CT data.^8^ This approach allows both datasets to be displayed as a combined rendering and provides a global overview of both abnormal PET uptake and its anatomic location.^8^

Prostate‐specific membrane antigen (PSMA) is a type II, transmembrane glycoprotein that is highly expressed on prostate cancer epithelial cells as well as the endothelium of tumour‐associated neovasculature in non‐prostate cancers.^9^ To date, PET agents targeting PSMA have primarily been used to image patients with prostate cancer at the time of initial staging^10^ and upon biochemical recurrence.^11^ However, PSMA PET radiotracers also have high sensitivity and specificity for identifying sites of other cancers, such as clear cell renal cell carcinoma.^12^, ^13^, ^14^

We performed cinematic rendering of a PET/CT performed with the PSMA‐targeted radiotracer ^18^F‐DCFPyL of a woman with oligometastatic clear cell renal cell carcinoma (Figure 1). The patient had radiotracer uptake in a discrete lesion in the left breast, which was highly conspicuous on the rendered images. In this case, the use of cinematic rendering allowed for the rapid identification and precise anatomical localization of the patient's site of disease. Although demonstrative of the potential of this reconstructive method for visualizing PET/CT data, further efforts are needed to define the role of cinematic rendering in clinical practice.

**FIGURE 1:**
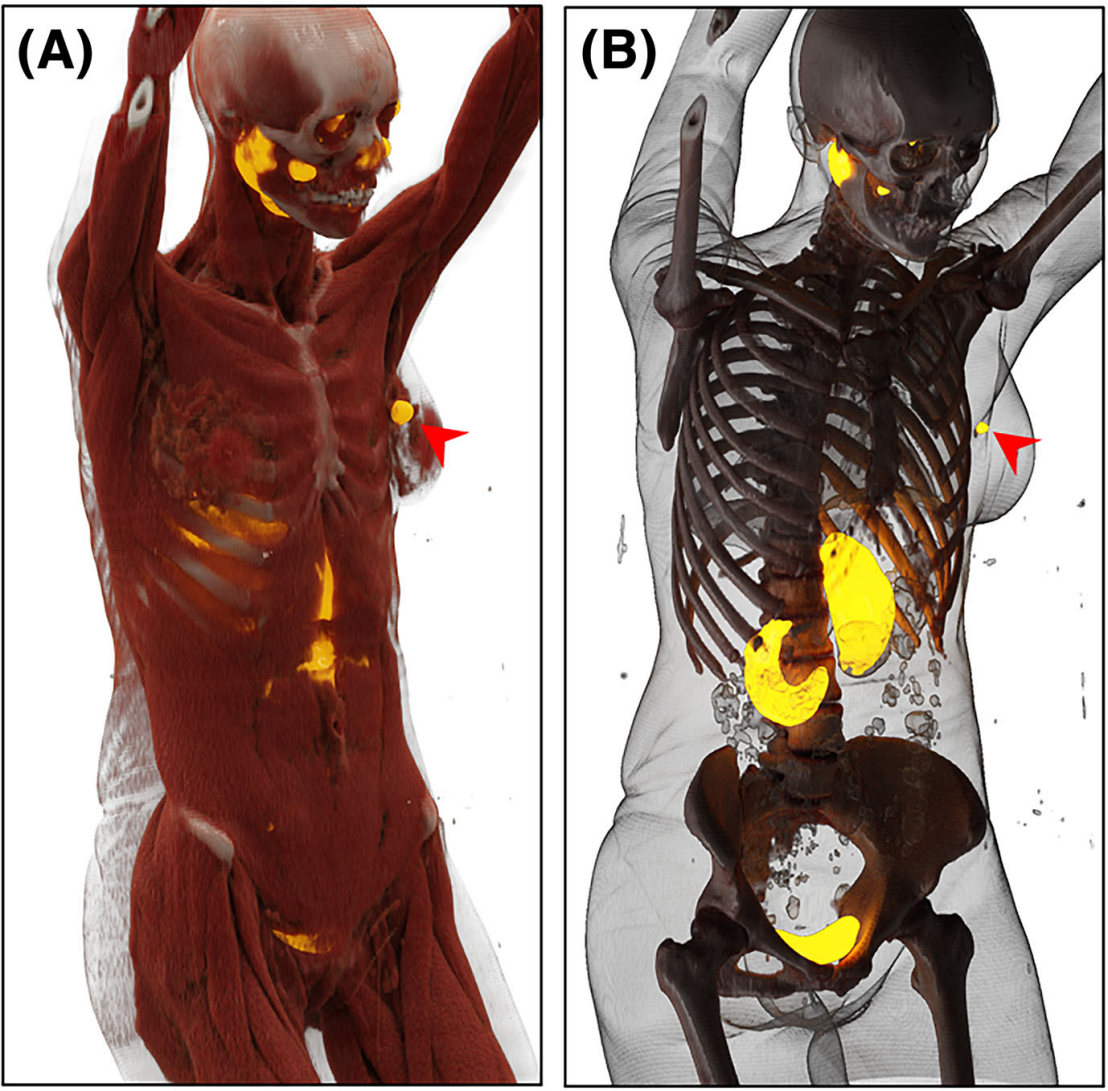
Cinematic rendering of an ^18^F‐DCFPyL PET/CT performed for a woman with oligometastatic clear cell renal cell carcinoma. (A) Musculoskeletal and (B) PET preset images showed intense radiotracer uptake in a biopsy‐proven metastasis to the patient's left breast (arrows). The internal lighting model used allowed fora high degree of contrast between the fused CT and PET images.

## AUTHOR CONTRIBUTIONS

SPR wrote the original draft and assisted with image analysis. SK developed the software application that was used. MAG led patient accrual and assisted with image analysis. EKF led image analysis and created the cinematic rendered images. SK, MAF, and EKF all critically revised the manuscript.

## CONFLICT OF INTEREST STATEMENT

EKF receives research support from Siemens and GE Healthcare and is a co‐founder and stockholder in HipGraphics, Inc. SPR and MAG have received research funding from Progenics Pharmaceuticals, Inc., a wholly owned subsidiary of Lantheus Pharmaceuticals, Inc., the licensee of ^18^F‐DCFPyL. SPR serves as a consultant to Progenics Pharmaceuticals, Inc. MAG has served as a consultant to Progenics Pharmaceuticals, Inc. SK is an employee of Siemens Healthineers.
